# The Association of Atherogenic Indices with Coronary Slow Flow: Evidence from a Large Cohort Study

**DOI:** 10.3390/diagnostics16050717

**Published:** 2026-02-28

**Authors:** Muzaffer Bayhatun, Sadettin Selçuk Baysal

**Affiliations:** Cardiology Department, Başakşehir Çam and Sakura City Hospital, 34480 İstanbul, Türkiye; sselcukbaysal@hotmail.com

**Keywords:** coronary slow flow, atherogenic dyslipidemia, atherogenic indices, Castelli risk indices, non-HDL cholesterol

## Abstract

**Background:** Coronary slow flow (CSF) is a microvascular disorder characterized by delayed perfusion despite the absence of significant epicardial stenosis. Although its exact pathophysiology remains unclear, endothelial dysfunction, oxidative stress, and atherogenic dyslipidemia have been implicated. Traditional lipid parameters may not fully capture the atherogenic burden, whereas atherogenic indices such as the atherogenic index of plasma (AIP), atherogenic coefficient (AC), and Castelli risk indices (CRI-I and CRI-II) may provide better predictive value. This study aimed to investigate the association between atherogenic indices and CSF in a large real-world angiographic cohort. **Methods:** This retrospective study included 25,486 patients who underwent coronary angiography between September 2020 and June 2024. A total of 464 patients with CSF (diagnosed by TIMI frame count criteria) and 408 controls with normal coronary flow (NCF) were identified. Atherogenic indices, including AIP, AC, CRI-I, CRI-II, and non-HDL cholesterol (non-HDL-C), were calculated. Multivariate logistic regression analysis identified independent predictors of CSF, while receiver operating characteristic (ROC) curve analysis was used to assess the diagnostic performance of each lipid-related parameter. **Results:** Patients with CSF had significantly higher AIP, AC, non-HDL-C, and CRI indices and lower HDL-C levels compared to controls (all, *p* < 0.05). Multivariate analysis identified AIP (OR: 1.73, 95% CI: 1.18–2.44, *p* = 0.004), age (OR: 1.02, 95% CI: 1.01–1.06, *p* = 0.014) and smoking (OR: 2.22, 95% CI: 1.36–2.84, *p* = 0.003) as independent predictors of CSF. ROC analysis showed modest but statistically significant discriminatory capacity for AIP (cut-off: 0.50; AUC: 0.629; 95% CI: 0.591–0.667; *p* < 0.001). AIP also demonstrated a weak yet significant correlation with mean TIMI frame count (rho = 0.245, *p* < 0.001), suggesting a potential link to microvascular dysfunction. **Conclusions:** Among the evaluated atherogenic indices, only AIP demonstrated an independent association with CSF. Despite modest discriminative performance that does not support standalone clinical prediction, AIP may reflect an underlying metabolic phenotype associated with CSF and serve as a complementary marker alongside traditional risk assessment. These findings should be interpreted as hypothesis-generating and warrant prospective validation.

## 1. Introduction

Coronary slow flow (CSF) is a clinically relevant angiographic phenomenon reflecting coronary microvascular dysfunction, characterized by the delayed passage of radiopaque contrast media into distal vascular structures despite the absence of significant stenosis in the epicardial coronary arteries during angiography [1,2,3]. While its pathophysiology remains incompletely understood, emerging evidence suggests that diffuse subclinical atherosclerosis, endothelial dysfunction, increased platelet aggregability, and microvascular tone abnormalities may contribute to its development [1,3,4,5,6,7]. Epidemiological studies have highlighted that male sex, smoking, and reduced high-density lipoprotein cholesterol (HDL-C) levels are more prevalent among patients with CSF [1]. Despite its distinct presentation, the relationship between CSF and traditional cardiovascular risk factors warrants further investigation, particularly in the context of atherogenic dyslipidemia and metabolic risk phenotypes.

Atherogenic dyslipidemia, a significant contributor to atherosclerosis, is characterized by elevated triglycerides (TG), small dense low-density lipoprotein cholesterol (LDL-C), and decreased HDL-C levels [8]. These lipid abnormalities disrupt endothelial function and promote atherogenesis through oxidative stress, inflammatory cytokine release, and impaired nitric oxide synthesis [9,10,11]. The atherogenic index of plasma (AIP), along with Castelli risk indices I and II (CRI-I and CRI-II) and the atherogenic coefficient (AC), are advanced lipid indices that provide deeper insights into cardiovascular risk compared to traditional lipid parameters. Emerging evidence supports their potential association with coronary artery disease; however, their relationship with CSF remains insufficiently explored [12,13,14,15].

Recent advances in intravascular imaging and pathophysiological studies indicate that CSF may represent an early stage of coronary atherosclerosis involving both epicardial and microvascular components. Functional and structural alterations in the microcirculation may lead to increased microvascular resistance, disturbed or heterogeneous coronary shear stress patterns, and impaired myocardial perfusion, potentially contributing to the progression of atherosclerotic processes. Moreover, subclinical diffuse coronary atherosclerosis and endothelial dysfunction associated with CSF may exacerbate cardiovascular risk, emphasizing the need for improved understanding of underlying metabolic and vascular mechanisms [1,5,6,16].

Given the limited data on the relationship between atherogenic indices and CSF, this study aims to investigate the association of lipid profiles and advanced atherogenic markers, including AIP and CRI indices, and CSF in a large real-world angiographic cohort.

## 2. Methods

### 2.1. Study Population and Design

This retrospective real-world observational study was designed to evaluate metabolic phenotypes associated with coronary slow flow (CSF) within an unselected angiographic population, thereby reflecting routine clinical practice rather than a matched case–control framework. A total of 25,486 patients who underwent coronary angiography at Başakşehir Çam and Sakura Şehir Hospital between September 2020 and June 2024 were screened. Coronary angiography was performed to evaluate ischemia based on clinical symptoms or non-invasive diagnostic tests, such as exercise treadmill testing or myocardial perfusion scintigraphy. Among these patients, 1271 were identified with CSF based on the Thrombolysis in Myocardial Infarction (TIMI) frame count method [17]. After applying exclusion criteria, 464 patients formed the CSF group. For the control group, 408 individuals with normal coronary flow (NCF) and no evidence of significant stenosis on angiography were included. The control group consisted of patients who underwent coronary angiography during the same period and were found to have angiographically normal coronary arteries without evidence of CSF. Controls were selected by simple random sampling from eligible cases after applying predefined inclusion and exclusion criteria, resulting in unequal group sizes consistent with the real-world observational design. Instead of matching, potential confounders were addressed using multivariable statistical adjustment. All participants in both groups were evaluated using the same procedural and diagnostic criteria. The study protocol was approved by the local Ethics Committee and adhered to the Declaration of Helsinki principles.

Diabetes mellitus (DM) was defined as fasting glucose ≥ 126 mg/dL, glycated hemoglobin (HbA1c) ≥ 6.5%, a prior diagnosis of DM, or the use of antidiabetic medications. Systolic blood pressure (SBP) ≥ 140 mmHg and/or diastolic blood pressure (DBP) ≥ 90 mmHg, or the use of antihypertensive therapy, were regarded as hypertension (HT). Dyslipidemia was defined as the use of lipid-lowering medications or total cholesterol (TC) ≥ 200 mg/dL, LDL-C ≥ 130 mg/dL, TG ≥ 150 mg/dL, HDL-C < 40 mg/dL in men and <50 mg/dL in women. Patients with a history of myocardial infarction, prior revascularization procedures (percutaneous coronary intervention or coronary artery bypass graft surgery), coronary ectasia, or coronary stenosis ≥ 50% were excluded. Additional exclusion criteria included cerebrovascular disease, renal dysfunction, left ventricular ejection fraction (LVEF) ≤ 50%, moderate-to-severe valvular heart disease, congenital heart disease, cardiomyopathies, hematological or thyroid diseases, and active inflammatory conditions. Patients receiving statins or any lipid-lowering therapy were excluded from the study to eliminate pharmacologic influence on lipid profiles and atherogenic indices. Data regarding demographics, clinical features, and laboratory parameters were retrieved from hospital records.

### 2.2. Coronary Angiography and TIMI Frame Count Analysis

Coronary angiography was performed using standard femoral or radial approaches and a Philips Azurion 7 system. Iopromide contrast agent (Bayer Pharma AG, Berlin, Germany) was used for visualization. Angiographic images were recorded at 30 frames per second, and TIMI frame counts (TFCs) were independently analyzed by two blinded cardiologists. Inter-observer and intra-observer variability for TFC measurements were assessed in a random subset of 50 patients using the intraclass correlation coefficient (ICC). The ICC for inter-observer reliability was 0.92 (95% CI: 0.88–0.95), indicating excellent agreement. Discrepancies were resolved by consensus.

The TFC method was applied to assess coronary flow. The first frame was defined as the frame where the contrast agent filled the coronary ostium, and the final frame was the one where the distal segment landmarks were visualized. To standardize TFC values for the left anterior descending artery (LAD), the corrected frame count was calculated by dividing the LAD TFC by 1.7. Patients were considered to have CSF if their TFC was two standard deviations above the mean reference values for at least one coronary artery (LAD: 36 ± 1, LCX (left circumflex artery): 22.2 ± 4, RCA (right coronary artery): 20.4 ± 3). Mean TFC values were derived by averaging the TFCs from LAD, LCX, and RCA for each patient [17].

### 2.3. Laboratory Analysis

Fasting blood samples were collected prior to angiography and analyzed using the Architect c8000 Chemistry System (Abbott Diagnostics, Abbott Park, IL, USA). Parameters measured included TC, TG, LDL-C, HDL-C, creatinine, and fasting glucose levels. LDL-C levels were directly measured using homogeneous enzymatic assays. Non-HDL cholesterol was calculated by subtracting HDL-C from total cholesterol (Non-HDL-C = TC − HDL-C), as recommended by current guidelines. Atherogenic indices were derived as follows: AIP = log10(TG/HDL-C), Castelli risk index I (CRI-I) = TC/HDL-C, Castelli risk index II (CRI-II) = LDL-C/HDL-C, and AC or non-HDL-C/HDL-C ratio (NHHR) = (TC − HDL-C)/HDL-C.

Complete blood count parameters, including white blood cell (WBC), neutrophil, and lymphocyte counts, were measured with a CELL-DYN Ruby hematology analyzer (Abbott Diagnostics, Abbott Park, IL, USA). Left ventricular function was assessed using transthoracic echocardiography, and LVEF was calculated using the Simpson method.

### 2.4. Statistical Analysis

All statistical analyses were performed using IBM SPSS Statistics version 26.0 and Python 3.11 (Scikit-learn and Statsmodels packages). Continuous variables were expressed as mean ± standard deviation or median (interquartile range) and categorical variables as counts and percentages. The Kolmogorov–Smirnov test was used to assess normality.

Group comparisons were conducted using Student’s *t*-test or the Mann–Whitney U test for continuous variables, and the chi-square or Fisher’s exact test for categorical variables, as appropriate. To account for multiple hypothesis testing in univariate comparisons (particularly of lipid and inflammatory markers), the Benjamini–Hochberg false discovery rate (FDR) correction was applied. All reported *p*-values in Table 1 and Table 2 reflect FDR-adjusted values.

Spearman correlation analysis was used to evaluate the association between mean TIMI frame count and atherogenic indices, including AIP, AC, CRI-I, CRI-II, and non-HDL-C. Binary logistic regression analysis was performed to identify independent predictors of coronary slow flow (CSF). Variables with *p* < 0.1 in univariate analyses and those deemed clinically relevant were considered for model inclusion. Multicollinearity among covariates was assessed using the variance inflation factor (VIF); values above 5 were considered indicative of collinearity, but all included variables had VIFs < 2.0.

Model calibration was evaluated with the Hosmer–Lemeshow goodness-of-fit test, and model discrimination was assessed using receiver operating characteristic (ROC) curve analysis. Area under the curve (AUC) values were reported with 95% confidence intervals, derived from 1000-sample bootstrap resampling. Optimal cut-off values for AIP and AC were identified using the Youden Index. Comparative model performance was evaluated by ROC analysis and calibration plots; however, ROC analyses were primarily interpreted to assess statistical discrimination rather than standalone clinical prediction performance.

A two-tailed *p*-value < 0.05 was considered statistically significant.

## 3. Results

A total of 464 patients with CSF and 408 control participants with NCF were included in the study. Overall, baseline clinical and laboratory characteristics were largely comparable between groups, with statistically significant yet modest absolute differences observed in selected metabolic and lipid-related parameters (Table 1). The mean ages of the CSF and NCF groups were 53 ± 10 years and 52 ± 7 years, respectively, with no significant difference between the groups (*p* = 0.781). Male predominance was observed in both groups, but the proportion of males was higher in the CSF group (64% vs. 60%, *p* = 0.311). Smoking prevalence was significantly higher in the CSF group compared to the NCF group (46% vs. 30%, *p* = 0.011). Other demographic and clinical characteristics, such as hypertension, DM, and body mass index (BMI), were comparable between the groups (all, *p* > 0.05).

Laboratory analysis revealed that non-HDL-C, AIP, AC, CRI-I, and CRI-II were significantly elevated in the CSF group compared to the NCF group (all, *p* < 0.05) (Table 2). In contrast, HDL-C levels were significantly lower in the CSF group (42.92 ± 11.72 mg/dL vs. 45.07 ± 12.56 mg/dL, *p* = 0.038). Additionally, inflammatory markers such as white blood cell count (WBC) and neutrophil count were significantly higher in the CSF group, while lymphocyte counts were significantly lower (all, *p* < 0.05). No significant differences were observed in TC, LDL, TG, creatinine and hemoglobin levels between the groups (all, *p* > 0.05). Although absolute differences in HDL-C and non-HDL-C levels between groups were modest, higher AIP values observed in the CSF group may reflect a shift toward a more pro-atherogenic lipoprotein profile not fully captured by traditional lipid parameters.

Angiographic evaluation showed significantly higher corrected TIMI frame counts for the LAD, LCX, and RCA arteries, as well as higher mean TFC values in the CSF group compared to controls (all *p* < 0.001). Among patients with CSF, 337 (72.6%) exhibited slow flow in the LAD, 312 (67.2%) in the LCX, and 331 (71.3%) in the RCA. CSF was observed in one vessel in 172 patients (37.1%), in two vessels in 128 patients (27.6%), and in all three vessels in 164 patients (35.3%).

**Figure 1:**
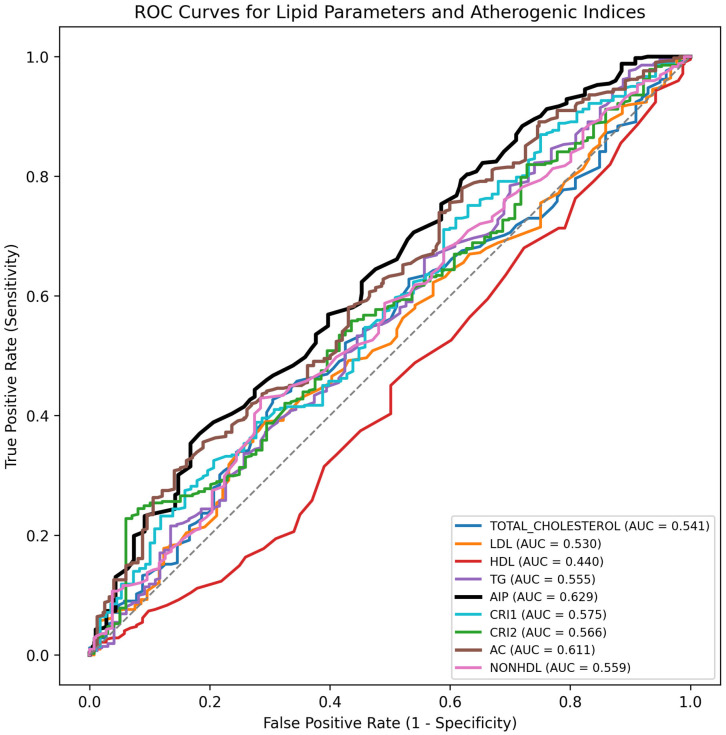
Receiver operating characteristic (ROC) curves for traditional lipid parameters and atherogenic indices in predicting coronary slow flow (CSF). ROC analysis was performed for total cholesterol, LDL-C, HDL-C, triglycerides (TG), non-HDL-C, atherogenic index of plasma (AIP), Castelli Risk Index I (CRI-I), Castelli Risk Index II (CRI-II), and atherogenic coefficient (AC). AIP and AC showed statistically significant but modest discriminatory ability (AUC = 0.629 and 0.611, respectively; both *p* < 0.001). The Youden Index was used to determine optimal cut-off values.

Receiver operating characteristic (ROC) curve analysis revealed an optimal cut-off value of 0.50 for AIP (AUC: 0.629; 95% CI: 0.591–0.667; sensitivity: 62.1%; specificity: 54.7%; *p* < 0.001) and 3.34 for AC (AUC: 0.611; 95% CI: 0.571–0.651; sensitivity: 60.7%; specificity: 52.9%; *p* < 0.001) (Table 3). Both indices demonstrated modest but statistically significant discriminatory capacity compared to traditional lipid markers such as LDL-C (AUC: 0.528) and HDL-C (AUC: 0.437) (Figure 1), indicating statistical association rather than strong standalone classification performance.

**Figure 2:**
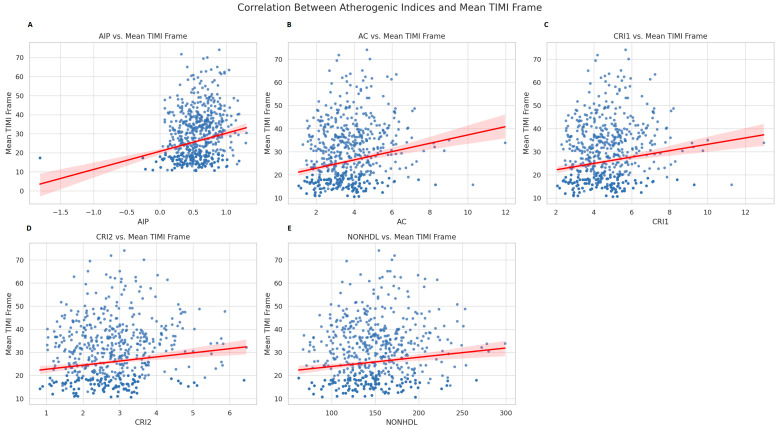
Correlation between mean TIMI frame count and atherogenic indices in patients with coronary slow flow. Scatter plots with Spearman correlation coefficients (ρ) and *p*-values are presented for the relationship between mean TIMI frame count and the following indices: (**A**) AIP (ρ = 0.245, *p* < 0.0001); (**B**) AC (ρ = 0.198, *p* < 0.0001); (**C**) CRI-I (ρ = 0.147, *p* < 0.0001); (**D**) CRI-II (ρ = 0.137, *p* = 0.0001); (**E**) Non-HDL-C (ρ = 0.120, *p* = 0.0006). Although the correlations are statistically significant, the strength of association is modest.

Correlation analysis revealed a statistically significant, though modest, positive correlation between mean TIMI frame and atherogenic indices. AIP showed the strongest association (rho = 0.245, *p* < 0.001), followed by AC (rho = 0.198), CRI-I (rho = 0.147), CRI-II (rho = 0.137), and non-HDL-C (rho = 0.120) (Figure 2).

**Figure 3:**
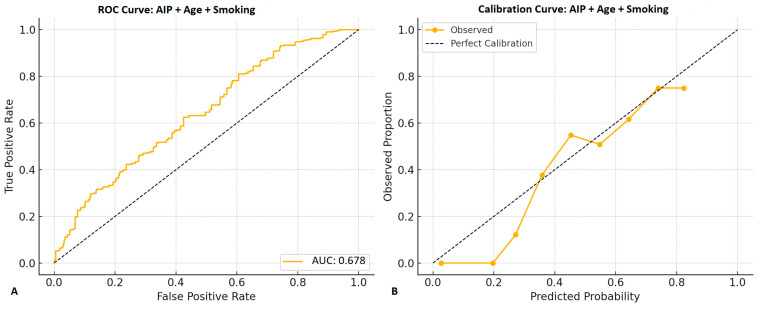
Receiver operating characteristic (ROC) curve of the multivariable model including AIP, age and smoking status for predicting coronary slow flow. The model achieved an area under the curve (AUC) of 0.678 (95% CI: 0.642–0.713), indicating modest discriminative performance. Bootstrap resampling (n = 1000) was used to validate the AUC. The model also demonstrated good calibration by the Hosmer-Lemeshow test (χ^2^ = 8.738, *p* = 0.365). (**A**) ROC curve, (**B**) Calibration curve.

In multivariate logistic regression analysis, a model including AIP, age, and smoking demonstrated stable performance and interpretability. Among the lipid-derived indices, only AIP was retained in the final model after assessment of multicollinearity and model parsimony, as other composite indices demonstrated substantial overlap and did not provide incremental explanatory value. All three variables were independently associated with CSF: AIP (OR: 1.73, 95% CI: 1.18–2.44, *p* = 0.004), age (OR: 1.02, 95% CI: 1.01–1.06, *p* = 0.014), and smoking (OR: 2.22, 95% CI: 1.36–2.84, *p* = 0.003) (Table 4). The model showed acceptable discrimination (AUC: 0.678; 95% CI: 0.642–0.713, bootstrap validated) and satisfactory calibration (Hosmer–Lemeshow χ^2^ = 8.738, *p* = 0.365) (Figure 3), supporting internal consistency without implying independent clinical prediction utility.

## 4. Discussion

This study investigated the relationship between CSF and various atherogenic parameters, including AIP, AC, CRI-I, and CRI-II. Our findings demonstrated that patients with CSF exhibited significantly elevated levels of atherogenic indices and non-HDL-C compared to controls. Furthermore, AIP, age, and smoking were independently associated with CSF. These findings support a potential link between atherogenic dyslipidemia, subclinical atherosclerosis, and coronary microvascular dysfunction, consistent with previous investigations, while leveraging a large real-world angiographic cohort to enhance statistical robustness [13,14]. However, although statistically significant associations were observed, the magnitude of discriminatory performance and absolute intergroup differences was modest, suggesting that these findings should be interpreted as reflecting subtle biological trends rather than strong standalone clinical predictors.

**Figure 4:**
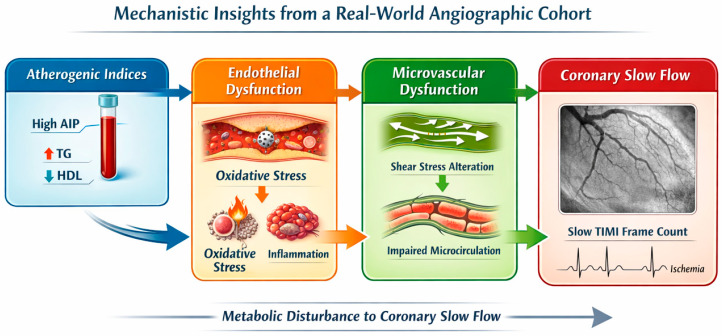
Proposed mechanistic pathway linking atherogenic dyslipidemia to coronary slow flow (CSF). Conceptual schematic illustrating the potential interactions between atherogenic lipid profiles (e.g., elevated AIP and related indices), endothelial dysfunction, oxidative stress, inflammation, and altered wall shear stress contributing to coronary microvascular dysfunction and the development of CSF. Atherogenic dyslipidemia may promote endothelial injury through metabolic and inflammatory pathways, while disturbed hemodynamic conditions further exacerbate vascular dysfunction. The dashed arrows indicate hypothesis-generating pathways derived from current evidence and supported by findings from the present real-world angiographic cohort.

CSF, characterized by delayed distal coronary perfusion without overt stenosis, has been increasingly associated with early-stage coronary atherosclerosis, endothelial dysfunction and microvascular abnormalities [1,4,5,6]. Advanced imaging modalities, such as intravascular ultrasonography (IVUS) and optical coherence tomography (OCT), have detected diffuse intimal thickening, vascular calcifications and lipid-laden plaques in CSF patients [18,19]. These findings support the hypothesis that CSF represents a preclinical stage of atherosclerosis, characterized by structural changes in the coronary microvasculature and epicardial arteries. Endothelial dysfunction is another crucial factor implicated in the pathophysiology of CSF. Impaired endothelial function leads to reduced nitric oxide bioavailability, increased oxidative stress, and heightened microvascular resistance, all of which contribute to the slow flow phenomenon [6,20,21]. Specifically, structural alterations and reduced flow velocity in CSF patients may create a state of relatively low or heterogeneous wall shear stress. This hemodynamic environment has been associated with a pro-atherogenic and pro-inflammatory endothelial phenotype, potentially amplifying vascular dysfunction and microvascular resistance [16]. The observed association between AIP and CSF in our cohort is consistent with this framework, suggesting that atherogenic lipoprotein patterns may interact with adverse hemodynamic conditions at the endothelial level. The proposed mechanistic framework integrating metabolic, endothelial, and hemodynamic pathways underlying CSF is illustrated in Figure 4, providing a conceptual model linking atherogenic dyslipidemia to impaired coronary microvascular function. Additionally, elevated levels of inflammatory markers and adhesion molecules, including intercellular adhesion molecule-1 (ICAM-1) and vascular cell adhesion molecule-1 (VCAM-1), indicate a pro-inflammatory, dysfunctional vascular environment, further supporting the link between chronic inflammation and CSF [20,22,23].

Atherogenic dyslipidemia, marked by elevated TG, small dense LDL-C, and reduced HDL-C levels, is a critical contributor to endothelial dysfunction and atherosclerotic processes [12,15]. Recent guidelines have highlighted the importance of non-HDL-C as a comprehensive marker of atherogenic lipoproteins, surpassing LDL-C in predicting cardiovascular events [24,25]. Non-HDL-C encompasses all atherogenic particles, including VLDL-C, lipoprotein(a), and intermediate-density lipoproteins, and is now recommended as a primary therapeutic target [26]. This underscores the relevance of combining traditional lipid parameters into indices like AIP, AC, and CRI-II, which provide integrated metabolic information and may reflect underlying risk patterns even when clinical effect sizes appear modest. These parameters reflect the balance between pro- and anti-atherogenic lipid particles, offering a more nuanced understanding of cardiovascular risk [12,15]. For example, AIP, an indirect indicator of small dense LDL-C, provides insights into endothelial dysfunction and the severity of subclinical atherosclerosis. Similarly, CRI-II, which combines LDL-C and HDL-C values, has been shown to outperform individual lipid parameters in predicting cardiovascular events [27,28].

In this study, we observed significantly higher levels of AIP, AC, CRI-I, CRI-II, and non-HDL-C in patients with CSF compared to controls. AIP, an indirect marker of small dense LDL-C, showed the strongest individual association with CSF and was modestly correlated with mean TIMI frame count (rho = 0.245, *p* < 0.001). Although this correlation may suggest a potential link between dyslipidemia severity and microvascular impairment, it should be interpreted cautiously given the absence of direct endothelial or inflammatory biomarker data. Among the indices evaluated, AIP demonstrated the highest discriminative capacity in ROC analysis, with an AUC of 0.629 (95% CI: 0.591–0.667; *p* < 0.001) and an optimal cut-off value of 0.50. AC also showed significant but weaker discrimination (AUC: 0.611; 95% CI: 0.571–0.651; *p* < 0.001) and was not retained in the final multivariable model. When interpreted alongside traditional lipid markers, the modestly higher AUC observed for AIP compared with LDL-C or HDL-C suggests that composite lipid indices may capture subtle metabolic interactions not fully reflected by individual lipid parameters. The retention of AIP as the sole lipid-derived variable in the final model likely reflects overlap among composite lipid indices and shared variance between related lipid parameters, with AIP providing the most parsimonious representation of atherogenic lipid imbalance after adjustment. These findings are partially consistent with earlier reports by Afşin et al. and Toprak et al., who reported higher AIP and AC thresholds with greater sensitivity and specificity [13,14]. The differences in cut-off values and predictive performance may be due to variations in study design, population characteristics, and lipid distributions.

In addition to lipid parameters, smoking demonstrated one of the strongest independent associations with CSF in our study. This observation is consistent with prior reports by Sanghvi et al. and Arbel et al., who identified smoking as an independent risk factor for CSF [29,30]. Compared with lipid-derived indices, the relatively stronger effect size observed for smoking may reflect direct vascular toxicity rather than indirect metabolic modulation. Mechanistically, chronic tobacco exposure promotes endothelial injury through increased reactive oxygen species generation, reduced nitric oxide bioavailability, and impaired endothelium-dependent vasodilation, all of which may increase microvascular resistance and contribute to delayed coronary flow. Smoking has also been associated with vascular inflammation, platelet activation, and altered endothelial responsiveness to shear stress, potentially amplifying susceptibility to coronary microvascular dysfunction [30]. These mechanisms reinforce the multifactorial nature of CSF, where atherogenic dyslipidemia, oxidative stress, inflammatory activation, and environmental vascular injury converge to drive microvascular impairment.

Beyond traditional lipid abnormalities, our findings may reflect deeper pathophysiological processes consistent with emerging models of coronary microvascular dysfunction. The modest but statistically significant correlations between atherogenic indices (especially AIP) and mean TIMI frame count suggest a potential link between lipid-driven endothelial injury and impaired microvascular perfusion. AIP, which is associated with small dense LDL particles and oxidative stress, may indicate heightened lipotoxicity at the microvascular level. Smoking, an independent predictor in our model, further exacerbates endothelial dysfunction via increased reactive oxygen species and reduced nitric oxide bioavailability. Moreover, elevated leukocyte and neutrophil counts and decreased lymphocyte levels in CSF patients suggest a pro-inflammatory milieu, which aligns with evidence of immune-mediated microvascular injury. Beyond absolute leukocyte counts, these findings may reflect an imbalance between innate and adaptive immune responses, a pattern commonly observed in chronic low-grade vascular inflammation within the coronary microvascular environment. Elevated neutrophil predominance together with relative lymphopenia has been linked to endothelial dysfunction, increased oxidative stress, and impaired microvascular regulation in cardiovascular disease. Although derived inflammatory indices such as the neutrophil-to-lymphocyte ratio (NLR) or systemic immune-inflammation index were not formally calculated in the present study, the observed leukocyte profile is consistent with an activated immune-inflammatory phenotype that may synergize with atherogenic lipid patterns to promote coronary microvascular dysfunction. However, as direct measurements of endothelial function or inflammatory biomarkers were not included, these mechanistic interpretations remain hypothetical and should be interpreted with caution.

From a clinical perspective, these findings may encourage clinicians to reconsider CSF not merely as an angiographic curiosity but as a potential manifestation of coronary microvascular disease. After identification of CSF in the catheterization laboratory, metabolic indices such as AIP may assist in phenotypic characterization and risk-oriented evaluation rather than serving as independent diagnostic tools. Additionally, because this study population was derived from patients undergoing coronary angiography, potential referral and indication biases should be considered when interpreting generalizability.

In summary, this study highlights that atherogenic indices—particularly AIP—may have a modest yet meaningful role in the risk stratification of patients with CSF. Although these indices alone do not offer high discriminatory power, they provide incremental insight when combined with conventional risk factors. Further prospective studies with mechanistic endpoints are warranted to determine whether targeting atherogenic dyslipidemia can improve coronary microvascular function and long-term outcomes in CSF patients.

## 5. Study Limitations

This study has several limitations that should be acknowledged. First, due to its retrospective and single-center design, the findings may not be fully generalizable to broader populations. The cohort consisted solely of Turkish patients, and ethnic or regional differences in lipid profiles, genetic predisposition, and environmental factors may influence the observed associations between atherogenic indices and coronary slow flow (CSF). Furthermore, because the study population was derived from patients undergoing coronary angiography, referral and indication bias cannot be excluded, and the findings may not directly reflect community-based populations.

Second, although our analysis included a range of lipid-derived atherogenic indices, we did not directly compare their predictive value to traditional lipid parameters (e.g., LDL-C, HDL-C, non-HDL-C) using reclassification metrics such as net reclassification improvement (NRI) or integrated discrimination improvement (IDI). The absence of these methods limits our ability to quantify the incremental predictive value of AIP over traditional lipid parameters. Future studies should incorporate these metrics to better assess the clinical utility of composite lipid markers. Moreover, other potentially informative biomarkers such as small dense LDL-C and apolipoprotein B (apoB) were not measured, which may have further improved risk stratification and provided a more comprehensive evaluation of atherogenic burden.

Third, although subgroup analyses stratified by sex, age group, or the number of vessels involved could have provided additional insight into potential heterogeneity of effects, such analyses were not performed. This decision was based on concerns regarding insufficient statistical power in subgroup strata and the risk of model overfitting. Future studies employing matched designs or adequately powered stratified analyses may help clarify subgroup-specific associations.

Fourth, while our findings suggest a role for endothelial dysfunction and inflammation in CSF pathogenesis, we did not include specific inflammatory markers such as high-sensitivity C-reactive protein (hsCRP) or interleukin-6 (IL-6), nor did we perform functional vascular assessments such as flow-mediated dilation (FMD). Therefore, mechanistic interpretations linking atherogenic dyslipidemia to coronary flow alterations remain hypothesis-generating rather than definitive.

Fifth, while control participants were randomly selected from patients with angiographically normal coronary arteries, no formal matching was performed for potential confounders such as dietary habits, physical activity, or socioeconomic status. Therefore, selection bias or residual confounding cannot be entirely excluded.

Finally, although multivariate analyses were methodologically robust, the modest discriminative performance of AIP suggests that it should be interpreted alongside established risk factors rather than as an isolated predictor. As with any observational study, causal relationships cannot be established. Prospective multicenter studies are needed to further clarify the clinical relevance of atherogenic indices in CSF.

## 6. Conclusions

Among the atherogenic indices evaluated, the atherogenic index of plasma (AIP) was independently associated with coronary slow flow (CSF), suggesting a potential link between atherogenic dyslipidemia and coronary microvascular dysfunction. Although the observed discriminative performance was modest, AIP may provide complementary metabolic insight beyond traditional lipid parameters and contribute to phenotypic characterization rather than serving as a standalone predictive tool. These findings support the concept that CSF may reflect an underlying metabolic–microvascular continuum rather than an isolated angiographic phenomenon. Future prospective studies incorporating mechanistic assessments, broader biomarker panels, and advanced risk stratification approaches are warranted to determine whether targeting this dyslipidemic phenotype can improve coronary microvascular function and long-term clinical outcomes.

## Figures and Tables

**Table 1:** Clinical, laboratory and angiographic data of the study population.

	CSF (*n* = 464)	NCF (*n* = 408)	*p*
Clinical data			
Age, years	53 ± 10	52 ± 7	0.781
Male, *n* (%)	300 (64)	248 (60)	0.311
BMI, kg/m^2^	27.1 ± 5.2	26.5 ± 4.3	0.288
Diabetes mellitus, *n* (%)	94 (20)	78 (19)	0.713
Hypertension, *n* (%)	163 (35)	132 (32)	0.452
Smoking, *n* (%)	218 (46)	122 (30)	0.011 *
Laboratory data			
Total cholesterol, mg/dL	192.96 ± 39.99	188.57 ± 37.86	0.069
LDL-C, mg/dL	113.62 ± 33.74	111.57 ± 33.49	0.162
HDL-C, mg/dL	42.92 ± 11.72	45.07 ± 12.56	0.038 *
Triglyceride, mg/dL	180.23 ± 58.45	168.38 ± 47.83	0.093
Non-HDL cholesterol, mg/dL	150.43 ± 39.20	142.14 ± 36.82	0.009 *
Creatinine, mg/dL	0.78 ± 0.14	0.76 ± 0.12	0.393
Hemoglobin, g/dL	14.21 ± 1.67	14.52 ± 1.44	0.452
WBC, 10^3^/mm^3^	7.88 ± 1.23	7.81 ± 1.38	0.031 *
Neutrophil, 10^3^/mm^3^	4.79 ± 0.78	4.71 ± 0.81	0.014 *
Lymphocyte, 10^3^/mm^3^	2.51 ± 1.88	2.55 ± 0.73	0.041 *
Platelet, 10^3^/mm^3^	234.96 ± 33.56	231.4 ± 38.97	0.749
TIMI frame count measurements			
LAD (corrected)	28.47 ± 10.34	11.80 ± 1.93	<0.001 *
Cx	35.70 ± 14.18	17.60 ± 3.52	<0.001 *
RCA	39.93 ± 17.50	18.76 ± 3.71	<0.001 *
Mean	34.70 ± 11.25	16.05 ± 2.54	<0.001 *

* Statistically significant (FDR-adjusted *p* < 0.05). Values are presented as mean ± standard deviation or number (%). BMI: body mass index, Cx: circumflex artery, HDL-C: high-density lipoprotein cholesterol, LAD: left anterior descending artery, LDL-C: low-density lipoprotein cholesterol, RCA: right coronary artery, WBC: white blood cell.

**Table 2:** Levels of atherogenic parameters of the study population.

	CSF (*n* = 464)	NCF (*n* = 408)	*p*
AIP	0.56 ± 0.26	0.47 ± 0.38	<0.001
CRI-I	4.76 ± 1.42	4.38 ± 1.33	<0.001
CRI-II	2.83 ± 0.99	2.59 ± 0.92	<0.001
AC	3.76 ± 1.42	3.38 ± 1.33	<0.001

Values are presented as mean ± standard deviation AC: Atherogenic coefficient, AIP: Atherogenic index of plasma, CRI: Castelli’s risk index.

**Table 3:** ROC Curve Analysis of Atherogenic Indices for Predicting Coronary Slow Flow.

Index	Cut-Off	AUC (95% CI)	Sensitivity (%)	Specificity (%)	*p*-Value
AIP	0.50	0.629 (0.591–0.667)	62.1	54.7	<0.001
AC	3.44	0.611 (0.571–0.651)	60.7	52.9	<0.001

AUC: Area under the curve; CI: Confidence interval; AIP: Atherogenic index of plasma; AC: Atherogenic coefficient.

**Table 4:** Multivariate regression analysis to determine the independent predictors of CSF.

	Odds Ratio	95% Confidence Interval	*p*
Smoking	2.22	1.36–2.84	0.003
AIP	1.73	1.18–2.44	0.004
age	1.02	1.01–1.06	0.014

Model performance: AUC = 0.678 (95% CI: 0.642–0.713, bootstrap validated). Hosmer–Lemeshow test: χ^2^ = 8.738, df = 8, *p* = 0.365. AIP: Atherogenic index of plasma. Logistic regression model includes AIP, age and smoking.

## Data Availability

The data presented in this study are available from the corresponding author upon reasonable request.
